# The Regulation of Tumor Cell Invasion and Metastasis by Endoplasmic Reticulum-to-Mitochondrial Ca^2+^ Transfer

**DOI:** 10.3389/fonc.2017.00171

**Published:** 2017-08-10

**Authors:** Carl White

**Affiliations:** ^1^Physiology and Biophysics, Chicago Medical School, Rosalind Franklin University of Medicine and Science, North Chicago, IL, United States

**Keywords:** migration, Bcl-2, Bcl-XL, MCL1, reactive oxygen species, voltage-dependent anion channel, STIM, Orai

## Abstract

Cell migration is one of the many processes orchestrated by calcium (Ca^2+^) signaling, and its dysregulation drives the increased invasive and metastatic potential of cancer cells. The ability of Ca^2+^ to function effectively as a regulator of migration requires the generation of temporally complex signals within spatially restricted microdomains. The generation and maintenance of these Ca^2+^ signals require a specific structural architecture and tightly regulated communication between the extracellular space, intracellular organelles, and cytoplasmic compartments. New insights into how Ca^2+^ microdomains are shaped by interorganellar Ca^2+^ communication have shed light on how Ca^2+^ coordinates cell migration by directing cellular polarization and the rearrangement of structural proteins. Importantly, we are beginning to understand how cancer subverts normal migration through the activity of oncogenes and tumor suppressors that impinge directly on the physiological function or expression levels of Ca^2+^ signaling proteins. In this review, we present and discuss research at the forefront of interorganellar Ca^2+^ signaling as it relates to cell migration, metastasis, and cancer progression, with special focus on endoplasmic reticulum-to-mitochondrial Ca^2+^ transfer.

## Introduction

Pathology is frequently associated with the dysregulation of intracellular calcium (Ca^2+^) signaling (1). Cancer is no exception, with many primary tumor cells and cell lines displaying aberrant expression of Ca^2+^ signaling genes (2, 3). While it is unlikely that somatic mutations affecting any one individual Ca^2+^ signaling gene are sufficient to drive tumorigenesis (4, 5), remodeling of the Ca^2+^ signal in cancer appears almost universal and confers survival advantages (3, 6). And so, it may be that dysfunctional Ca^2+^ signaling is indeed a determinant of tumorigenesis when coincident with cancer-driving oncogene and tumor suppressor mutations.

Also, many oncoproteins and tumor suppressor proteins can themselves directly modulate Ca^2+^ signaling. They achieve this, in large part, by interacting with Ca^2+^ channels, pumps, and exchangers localized at the plasma membrane and various intracellular compartments. The Bcl-2 family of oncoproteins has been the most extensively studied in this respect and found to regulate Ca^2+^ signaling in ways that complement their roles as apoptotic regulators, as recently reviewed (7). Similarly, oncogenic Ras (8, 9) and the tumor suppressors promyelocytic leukemia (PML) (10), p53 (11), and BRCA1 (12) can all regulate apoptosis by impinging on the Ca^2+^ signal. Many of these proteins are enriched in spatially restricted domains created by the close apposition between the endoplasmic reticulum (ER) and the mitochondria, known as mitochondria-associated membranes (MAMs), where they function to modulate the flow of Ca^2+^ from the ER to mitochondria.

Ca^2+^ signaling also plays a role in cancer cell invasion and metastasis. Several different plasma membrane and ER-localized Ca^2+^ channels regulate the activity of effectors involved in motility and adhesion. Most of this regulation occurs by modifying the cytoplasmic Ca^2+^ signal and has been reviewed previously (13, 14). The significance of ER-mitochondrial Ca^2+^ communication in invasion and metastasis, however, has only recently emerged. This review will assess the literature relating to ER-mitochondrial Ca^2+^ communication. Our goal is to outline a theoretical framework that mechanistically links cancer-driven changes in ER-mitochondrial Ca^2+^ communication to its invasive and metastatic properties. We have made every attempt to include and reference original studies specifically related to this topic. When discussing a well-established concept, however, we direct readers to an appropriate review article.

## Ca^2+^ Signaling Regulates Multiple Steps of the Invasion-Metastatic Cascade

Tumor metastasis directly accounts for the vast majority of cancer deaths (15). Metastasis is characterized by a sequence of events known as the invasion-metastatic cascade (16, 17). During this process, cancer cells lose their attachment to other cells and the extracellular matrix (ECM), acquire migratory capabilities and invade neighboring tissues by degrading and moving through the ECM, and ultimately transit to secondary sites by finding their way into the blood and lymphatic circulation. Importantly, Ca^2+^ signaling plays a key role at a number of points in the invasion-metastatic cascade.

### Adhesion and Epithelial–Mesenchymal Transition (EMT)

The invasion-metastatic cascade begins with the loss of cell–ECM and cell–cell adhesion. Cells are linked to the ECM at focal adhesion points by structural complexes connecting membrane spanning integrins to the cytoskeleton. And so, the rate of focal adhesion assembly and disassembly governs the cell’s migratory ability. The process of disassembly is Ca^2+^ sensitive and triggered by Ca^2+^ oscillations that promote the association of focal adhesion kinase (FAK), a regulator of focal adhesion turnover, with the focal adhesion complex (18). The Ca^2+^ oscillations, which are spatially restricted at the focal adhesions (19), increase the residency of FAK at these sites through Ca^2+^/calmodulin-dependent protein kinase II (CaMKII)-dependent regulation of its phosphorylation status (19–22).

The loss of cell–cell adhesion is also mediated through the process of EMT (23). The EMT process converts polarized epithelial cells into highly motile mesenchymal cells, defined by the induction of the mesenchymal markers N-cadherin, vimentin, and transcription factors, Snail, Slug, and Twist. Induction of EMT in the MDA-MB-231 breast cancer cell line was dependent on increased store-operated Ca2^+^ entry (SOCE) driven by expression of the SOCE proteins, stromal interaction molecule 1 (STIM1) and Orai1 (24). The SOCE pathway is activated in response to depletion of ER Ca^2+^ stores, which causes the ER-localized STIM to bind to and open the plasma membrane Ca^2+^ channel Orai (25). In contrast, SOCE was not important for EMT in MDA-MB-468 breast cancer cells (26). In these cells, the Ca^2+^ permeable transient receptor potential canonical type 1 channel was implicated as a sensitizer to EMT (26). Moreover, a subsequent study of MDA-MB-468 cells showed a requirement for Ca^2+^-permeable transient receptor potential melastatin-like 7 (TRPM7) channels in increasing vimentin expression through the signal transducer and activator of transcription 3 pathway (27). Collectively, these studies define a role for Ca^2+^ signaling in EMT and hint that EMT regulation by Ca^2+^ is likely to involve diverse mechanisms that are highly dependent on cell type and stimulus.

### Migration and ECM Degradation

In the second step of the invasion-metastatic cascade, freely migrating cancer cells invade the surrounding stromal tissue. Migrating cells move by a cyclical process that begins with the extension of leading edge protrusions, known as lamellipodia. Lamellipodia attach to the substratum and contraction of the trailing rear edge moves the cell toward the lamellipodia (28, 29). At the leading edge, local Ca^2+^ signals control forward movement by regulating lamellipodia retraction and adhesion cycling through the activation of actin filament contraction (30, 31). Actin dynamics can also be influenced more indirectly by Ca^2+^ signaling, through activation of Ca^2+^-dependent kinases (30, 32) and regulation of Rac1, RhoA, and Cdc42 GTPases (33–36). In addition to forward motion, directional steering is also dependent on spatially restricted Ca^2+^ signals. These events, termed “Ca^2+^ flickers,” are triggered by Ca^2+^ influx through TRPM7 channels and amplified by ER Ca^2+^ release *via* inositol 1,4,5-trisphosphate receptor type 2 activation (37). While these spatially localized Ca^2+^ fluxes play important roles at the leading edge, there is on average, a front-to-rear increase in Ca^2+^ concentration (38–40). At the trailing edge, Ca^2+^ signaling is determined, in large part, by Ca^2+^ influx through L-type Ca^2+^ channels, which serves to maintain contractility and stabilize directional movement (40).

Invasive cancer cells migrate through their surrounding tissue. They do this by proteolytically degrading the ECM with enzymes that include matrix metalloproteinases (MMPs) and cathepsins (41). Importantly, Ca^2+^ influx determines how these enzymes influence metastasis. In a prostate cancer cell study, the metastatic potential was dependent on the expression of MMP2, MMP9, and cathepsin B regulated by Ca^2+^ influx through the transient receptor potential melastatin 2 (TRPV2) channel (42). Another study identified a role for SOCE (43). Activation of STIM/Orai was shown to influence melanoma metastasis by maintaining levels of membrane type1 MMP (MT1-MMP) at the plasma membrane (43).

## Involvement of ER-Mitochondrial Ca^2+^ Flux in Cancer Cell Invasion and Metastasis

**Figure 1:**
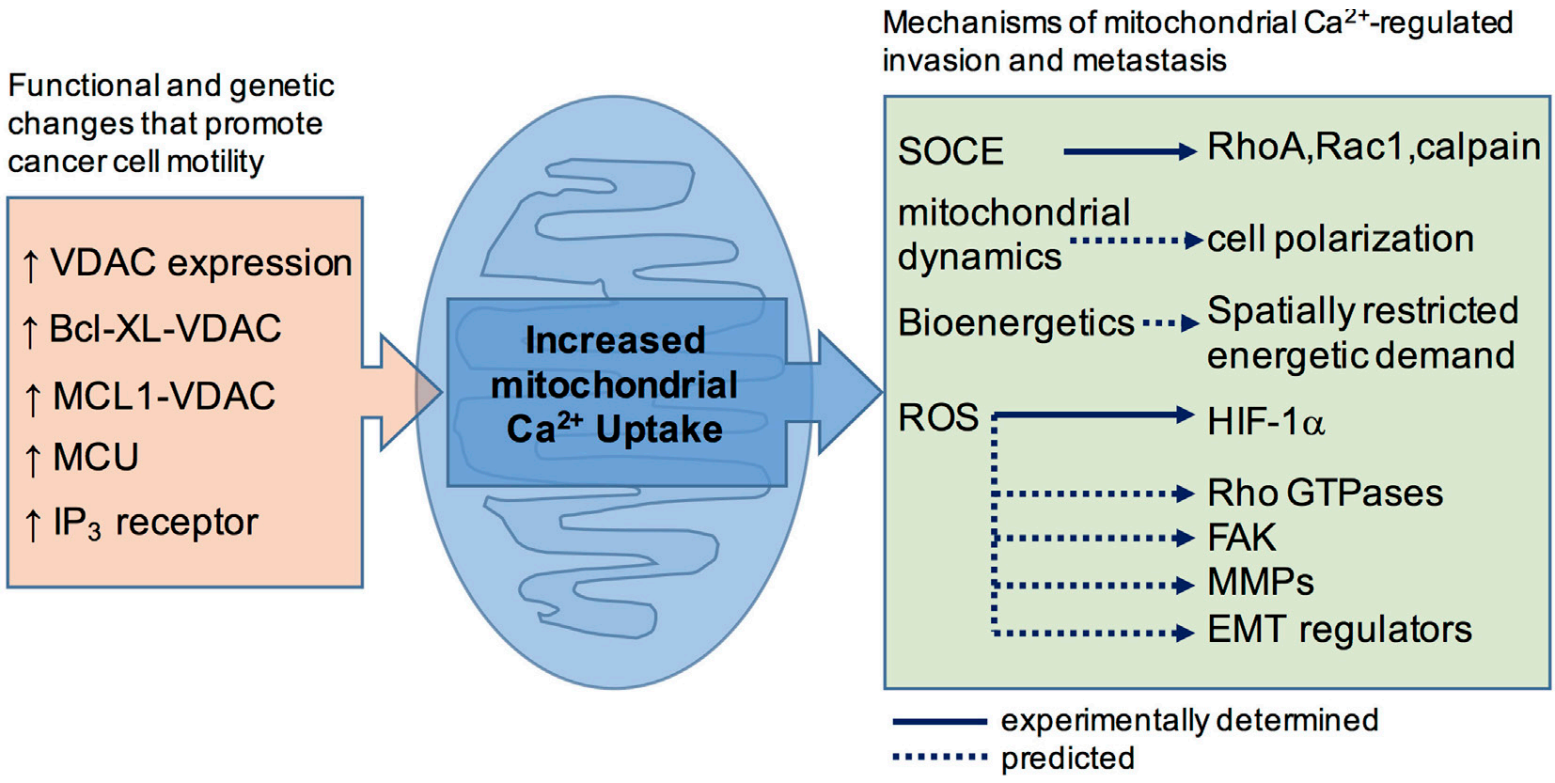
A schematic summary showing how cancer-associated functional and genetic changes that promote mitochondrial Ca^2+^ uptake are linked, or are predicted to link, to motility effector.

Cancer causes transcriptional and functional changes that often affect regulators of cytoplasmic Ca^2+^, including the TRP channels and components of STIM/Orai-mediated Ca^2+^ entry, for reviews see Ref. (2, 13, 14, 44). These changes are likely to have the greatest impact on the spatiotemporal profile of the cytoplasmic Ca^2+^ signal and affect the invasion-metastatic cascade by impinging on the cytoplasmically localized effectors outlined above (22, 45). As alluded to earlier, cancer is also associated with altered mitochondrial Ca^2+^ handling, brought about by changes in the expression profile of the mitochondrial Ca^2+^ uptake machinery, as well as oncoproteins and tumor suppressors at the MAM (46). Recent investigations using primary tumor models and cancer cell lines support the concept that the survival advantages of altered mitochondrial Ca^2+^ derive from effects on cellular metabolism (47) and apoptosis sensitivity (48–52). In the following section, we assess the evidence that altered ER-mitochondrial Ca^2+^ is also a determinant of increased invasive and metastatic potential (Figure 1).

### Mitochondria-Associated Membrane

The ER-localized inositol 1,4,5-trisphosphate receptor (IP_3_R) and ryanodine receptor Ca^2+^ release channels deliver Ca^2+^ to the mitochondria (53–55). Structural elements, which include physical tethers linking both membranes (56, 57) and protein–protein interactions that bridge the ER release and mitochondrial uptake machinery (58, 59), facilitate the Ca^2+^ transfer. To get to the matrix, Ca^2+^ first moves across the outer mitochondrial membrane through the voltage-dependent anion channel (VDAC) (60–62). The VDAC is a porin channel and diffusion pathway for ions and metabolites (62). Despite its large pore size, VDAC can function as a highly regulated Ca^2+^ permeability (63, 64) that directly couples to IP_3_R-dependent Ca^2+^ release through interactions with the mitochondrial chaperone GRP75 (58). From the intermembrane space, Ca^2+^ moves across the inner membrane through the mitochondrial Ca^2+^ uniporter (MCU) (65, 66). Cancer remodels the MAM architecture by changing the expression levels of ER and mitochondrial Ca^2+^ channel proteins or their associated binding partners and regulators (46). Restructuring the MAMs and the resultant effects on mitochondrial Ca^2+^ homeostasis impinges on many processes including metabolism, bioenergetics, cell death, proliferation, mitochondrial dynamics, and cytoplasmic Ca^2+^ signaling. For the purpose of this review, we restrict our focus to those effects that most profoundly impact, or are likely to impact, invasion and metastasis.

### Voltage-Dependent Anion Channel

Expression of the VDAC1 isoform is robustly increased in many cancer cell types (67) and reliably predicts survival outcomes in breast, colon, and lung cancers (68, 69). Increased VDAC likely promotes cancer cell growth primarily by influencing mitochondrial metabolism and apoptosis (70–72), processes that are also tightly regulated by VDAC interactions with hexokinase and members of the Bcl-2 family (72–74). Importantly, VDAC1 knockdown reduced cancer cell migration *in vitro* and suppressed tumor growth *in vivo* (75, 76). A decrease in VDAC expression would be expected to limit mitochondrial Ca^2+^ uptake (61). Indeed, VDAC influences cell migration by a mechanism that involves the regulation of mitochondrial Ca^2+^ uptake by interactions with Bcl-2 family proteins. The structural determinants and functional correlates of the VDAC-Bcl-2 protein interactions have been well characterized, as reviewed in Ref. 67. Antiapoptotic Bcl-XL and MCL1 both bind to VDAC1 and VDAC3 isoforms to promote mitochondrial Ca^2+^ uptake and drive cell migration (77–80). Importantly, disrupting the Bcl-XL-VDAC and MCL1-VDAC interactions was found to inhibit migration of triple negative breast cancer cells (80) and non-small cell lung carcinoma cells, respectively (78). These data raise the possibility of suppressing invasion and metastasis by targeting VDAC-Bcl-2 protein interactions.

### Inositol 1,4,5-Trisphosphate Receptors

The type 3 IP_3_R isoform (IP_3_R-3), which is absent in normal colorectal mucosa, is expressed in colorectal carcinoma. Moreover, the expression is greatest at the invasive margin and strongly correlated with metastasis and patient survival (81). The IP_3_R-3 is also overexpressed in human glioblastoma tissue (82). Inhibiting IP_3_Rs in glioblastoma cell lines reduced invasion *in vitro*; it also reduced invasion *in vivo* and prolonged survival by suppressing tumor growth (82). These studies, however, did not define the mechanisms by which increased IP_3_R-3 expression directs invasion and metastasis. Interestingly, the IP_3_R-3 is enriched in at the MAM in some cell types (83), and it may preferentially deliver Ca^2+^ to the mitochondria under certain conditions (84). It is possible then that increased IP_3_R abundance promotes invasion and metastasis by increasing Ca^2+^ delivery to the mitochondria.

### Mitochondrial Ca^2+^ Uniporter

The MCU machinery includes the MCU pore-forming subunit (65, 66) or its dominant-negative MCUb (85), together with associated regulators EMRE (86, 87) and MICU1-3 (88–91). Analysis of gene expression databases revealed that MCU levels are increased in several subtypes of breast cancer and correlated with tumor size, invasive and metastatic indices, and patient survival (92–94). While expression changes in the MCU regulators MCU1-3 and EMRE did not correlate with tumor size and invasiveness (92), poorer patient survival did correlate with increased MCU in combination with decreased MICU1 (94). The involvement of MCU in cancer progression was demonstrated *in vivo* by Tosatto et al., who showed that breast cancer tumor xenografts derived from MCU-deleted cells grew more slowly and were less likely to metastasize (92). *In vitro* experiments that knocked-down or inhibited MCU in breast cancer cell lines decreased mitochondrial Ca^2+^ uptake to inhibit migration and invasive potential without affecting cell survival (92, 94), proliferation, or apoptosis (95).

## Mechanisms of Mitochondrial Ca^2+^-Regulated Invasion and Metastasis

### Store-Operated Ca^2+^ Entry (SOCE)

Subplasmalemmal mitochondria regulate the activation and inactivation properties of SOCE by buffering incoming Ca^2+^ (96–98). Activation of SOCE is dependent on ER Ca^2+^ depletion, suggesting that ER, SOCE, and mitochondria are functionally coupled. Indeed, by limiting Ca^2+^ accumulation around the mouth of the IP_3_R, mitochondrial Ca^2+^ uptake prevents Ca^2+^-dependent inactivation of IP_3_Rs, which further depletes ER Ca^2+^ stores to promote SOCE (99). Given the Ca^2+^ communication between ER, mitochondrial and SOCE pathways, it is not surprising that MCU knockdown in MDA-MB-231 (93) and Hs578t (95) breast cancer lines inhibited both mitochondrial Ca^2+^ accumulation and SOCE. In the Hs578t cells, this caused a loss of cell polarity and migration associated with decreased RhoA, Rac1, and calpain activities (95). In these experiments, inhibiting SOCE (93, 95) or chelating intracellular Ca^2+^ (95) recapitulated the effects of MCU knockdown on migration. These data are consistent with studies defining STIM and Orai as key players in regulating invasion and metastasis (43, 100, 101) and suggest that altered MCU expression in cancer cells can influence downstream motility effectors by regulating SOCE.

### Mitochondrial Dynamics

Mitochondria redistribute to the leading edge of cancer cells to support the increased bioenergetic demands at the invadopodia (102–105). Interestingly, the translocation of mitochondria to subplasmalemmal sites also plays a critical role during immune cell activation, where they regulate Ca^2+^ influx through SOCE (106). It is yet to be determined, however, if mitochondrial positioning, and its influence on Ca^2+^ influx, affects the polarization of cytoplasmic Ca^2+^ signaling in migrating cancer cells.

The translocation of mitochondria is dependent on increased mitochondrial fission, a process also promoted by mitochondrial Ca^2+^ accumulation (107, 108). Evidence that MCU plays a role in fission comes from the observation that fission is inhibited by the pharmacological block of the MCU (109, 110) and enhanced by loss-of-function mutations in MICU1, which promote mitochondrial Ca^2+^ uptake (111). Mechanistically, mitochondrial Ca^2+^ might influence fission by regulating the activity of dynamin-related protein 1 (Drp1). The ability of Drp1 to promote fission is dependent on phosphorylation at serine 616 (S616) and dephosphorylation of serine 637 (S637) (112, 113). Cytoplasmic Ca^2+^ signaling is known to regulate the phosphorylation status of Drp1 through calcineurin-dependent dephosphorylation of S637 (112, 114), and more recently it was found that blocking the MCU suppressed fission by decreasing Drp1 phosphorylation at S616 (115). These observations are relevant to this review because Drp1 is widely associated with tumor invasion and metastatic potential (104, 116–118), and increased S616 is found in breast cancer and lymph node metastases (104). Although speculative, it is possible that increased mitochondrial Ca^2+^ uptake in cancer cells links to invasion and metastasis through the processes of fission and mitochondrial localization.

### Bioenergetics

Mitochondrial Ca^2+^ activates several Ca^2+^-dependent enzymes involved in the tricarboxylic acid (TCA) cycle (119). Work by Cárdenas et al. showed that constitutive low-level ER-mitochondrial Ca^2+^ transfer maintains flux through the TCA cycle to fuel oxidative phosphorylation and ATP production (120). In a follow-up study, the same group showed that blocking IP_3_Rs in cancer cells impaired oxidative phosphorylation, which killed cells by necrosis and reduced tumor growth *in vivo* (47). Although not examined in these studies, one might expect a similarly invoked bioenergetic crisis to inhibit cancer cell invasion and metastasis. Such an outcome is predicated based on the requirement for functional oxidative phosphorylation in tumor metastasis (121, 122), as well as the spatially restricted bioenergetic demands needed for cell migration (102–105).

### Reactive Oxygen Species (ROS)

Mitochondrial ROS are generated as a consequence of normal respiration. As electrons supplied by the TCA cycle are passed down the electron transport chain, they escape, mostly at complex I and III, to react with O_2_ and produce ROS. Mitochondrial Ca^2+^ uptake can increase ROS production at complexes I, III and IV under a variety of conditions. Although the mechanisms are still unclear, a number of possibilities have been proposed, as reviewed previously (123, 124). Also, mitochondrial Ca^2+^ can promote the release of ROS accumulated in cristae and intermembrane spaces through Ca^2+^-dependent increases in matrix volume (125).

Increased mitochondrial ROS production is a known determinant of tumor growth and metastasis (126, 127) that likely drives invasion and metastasis by increasing cell migration (128–130). Importantly, increased migration is repeatedly correlated with increased ER-mitochondrial Ca^2+^ uptake (92–95). While excessive ROS production is toxic and excessive mitochondrial Ca^2+^ uptake inhibits rather than promotes migration (78, 131), physiological Ca^2+^-dependent ROS production is a major mitochondrially derived signal involved in regulating downstream effectors. In one study, increased ER-mitochondrial Ca^2+^ transfer in non-small cell lung carcinoma cells was caused by MCL1–VDAC interactions that promoted cell migration by increasing mitochondrial ROS production (78). In another study, increased mitochondrial Ca^2+^ uptake increased breast cancer cell xenograft growth and metastasis by increased ROS-dependent expression of HIF-1α (92). To our knowledge, HIF-1α is the only cell migration regulator that has been specifically linked to mitochondrial Ca^2+^-dependent ROS production. Nevertheless, many migration effectors are sensitive to ROS signaling (132). Perhaps more intriguingly, many of these, including the Rho GTPases, FAK, MMPs, and mediators of EMT, are sensitive to both ROS and Ca^2+^ signals, see Ref. (13, 132) for complete listings of ROS and Ca^2+^-sensitive targets, respectively. The degree of overlap between ROS and Ca^2+^-sensitive effectors highlights a need to carefully differentiate between ROS and Ca^2+^-dependent effects when probing the role of ER-mitochondrial Ca^2+^ transfer.

## Conclusion

The molecular identification of the SOCE and MCU machinery, the introduction of powerful molecular tools, and the evolution of cancer genetics have all contributed to developing our understanding of how Ca^2+^ signals regulate cancer cell invasion and metastasis. As we have seen, a picture has emerged in which ER Ca^2+^ release, mitochondrial Ca^2+^ uptake, and plasmalemmal Ca^2+^ influx work together to exquisitely regulate cell motility. This complexity, however, should not dissuade efforts to examine the possibility of therapeutically targeting ER-mitochondrial Ca^2+^ transfer to affect metastasis. Encouragingly, many of the studies reviewed here have already demonstrated the feasibility of such an approach, showing reduced metastasis *in vivo* after targeting IP_3_Rs (47), STIM/Orai (22, 45), or MCU (92). In addition, therapeutics originally designed to promote cell death might also be useful for limiting metastasis. In this case, the Bcl-2 inhibitors, both BH3 and BH4 mimetics (80, 133), as well as recently developed MCL1 inhibitors (134), would be expected to suppress cell migration by limiting ER-mitochondrial Ca^2+^ transfer.

## Author Contributions

CW wrote the manuscript and designed and prepared figures.

## Conflict of Interest Statement

The author declares that the research was conducted in the absence of any commercial or financial relationships that could be construed as a potential conflict of interest.
